# Two Pentaplex Real-Time Fluorescent PCR Assays for Simultaneous Detection of Ten Ovine Respiratory and Reproductive Pathogens

**DOI:** 10.3390/v18091003

**Published:** 2026-09-11

**Authors:** Suqiu Wang, Zilong Bai, Benyi Wan, Tong Ren, Wei Zhang, Liwei Xu, Zhiqiang Gao, Congliang Deng, Sile Du, Dan Wu, Jing Pu, Xiangpeng Zhao, Jiawei Zhang, Quanguo Liu, Lin Wang, Ke Liu, Xiju Shi

**Affiliations:** 1 Science and Technology Research Center of China Customs, Beijing 100026, China; 2 Shanghai Veterinary Research Institute, Chinese Academy of Agricultural Science, No. 518, Ziyue Road, Shanghai 200241, China

**Keywords:** pentaplex real-time fluorescent PCR, simultaneous detection, ovine pathogens

## Abstract

We developed and evaluated two pentaplex real-time fluorescent PCR assays (Panel 1/Panel 2) for simultaneous detection of ten ovine pathogens. Panel 1 targets respiratory pathogens: Bluetongue virus (BTV), Foot-and-mouth disease virus (FMDV), Schmallenberg virus (SBV), Peste des petits ruminants virus (PPRV), and Sheep pox virus-Goat pox virus (SPPV-GTPV). Panel 2 targets reproductive pathogens: Border disease virus (BDV), *Brucella* spp. (Bru), Toxoplasma gondii (*T. gondii*), Chlamydia abortus (*C. abortus*), and Caprine arthritis-encephalitis virus (CAEV). Each panel uses six fluorescent channels for detection. Validation using reference pathogens and plasmids confirmed that the assay can effectively detect the target pathogens. The Limit of Detection at 95% probability (LOD95) ranged from 2.9 to 6.1 copies/μL for all targets, with correlation coefficients (R^2^) greater than 0.99. The intra-assay and inter-assay coefficients of variation (CV) were less than 1.5% at both high (10^5^ copies/μL) and low (10^3^ copies/μL) template concentrations. Cross-reactivity tests demonstrated high specificity with no cross-detection among different targets. Validation using 620 clinical samples collected from multiple provinces in China showed strong concordance with reference methods. This multiplex assay provides a rapid, sensitive, specific, and reliable tool for the differential detection and surveillance of major ovine respiratory and reproductive pathogens.

## 1. Introduction

Sheep and goats are economically significant livestock species globally, and their health management is critical for sustainable animal husbandry [1]. A variety of pathogens, including viruses, bacteria, and parasites, pose significant threats to small ruminant populations, causing substantial economic losses and impeding the development of the sheep industry. Among these, Bluetongue virus (BTV) is an Orbivirus transmitted by Culicoides midges, causing bluetongue disease characterized by fever, facial edema, and hyperemia of mucous membranes [2]. Foot-and-mouth disease virus (FMDV) leads to vesicular lesions in cloven-hoofed animals [3,4]. Schmallenberg virus (SBV), an emerging Orthobunyavirus, causes congenital malformations in newborns [5,6]. Peste des petits ruminants virus (PPRV) is the causative agent of peste des petits ruminants [7], a highly contagious disease with mortality rates up to 90%. Sheep pox virus and Goat pox virus (SPPV-GTPV), belonging to the genus Capripoxvirus [8,9], cause systemic febrile symptoms and characteristic pox lesions in sheep and goats. In addition, Border disease virus (BDV) causes border disease in lambs [10], while *Brucella* spp. are zoonotic bacteria responsible for brucellosis [11,12]. Toxoplasma gondii (*T. gondii*) is a widespread protozoan parasite causing toxoplasmosis [13]. Chlamydia abortus (*C. abortus*) and Caprine arthritis-encephalitis (CAEV) are pathogen infections leading to chronic inflammatory diseases [14,15,16].

Rapid and accurate laboratory diagnosis is essential for effective disease control and prevention. Conventional diagnostic methods, including virus isolation, bacterial culture, and serological assays, are time-consuming, labor-intensive, or insufficient for early infection detection. Polymerase chain reaction (PCR) and its derivatives have become the gold standard for pathogen detection due to their high sensitivity, specificity, and speed [17,18]. Multiplex real-time fluorescent PCR, which allows simultaneous detection of multiple targets in a single reaction, is particularly advantageous for syndromic panel testing and differential diagnosis.

In this study, we developed and evaluated a pentaplex real-time fluorescent PCR assay (Panel 1/Panel 2) for the simultaneous detection of Panel 1 (respiratory pathogens): BTV, FMDV, SBV, PPRV, and SPPV-GTPV; and Panel 2 (reproductive pathogens): BDV, Bru, *T. gondii*, *C. abortus*, and CAEV. These ten pathogens were selected based on three criteria. (i) significant economic impact on the Chinese sheep industry; (ii) inclusion in the World Organisation for Animal Health (WOAH) notifiable disease list or national quarantine regulations; and (iii) the current lack of a unified multiplex molecular detection platform covering both respiratory and reproductive syndromes. BTV has been detected in multiple Chinese provinces since 2014. FMDV remains endemic in certain regions. SBV, although not yet reported in China, represents a significant emerging threat due to its rapid spread across Europe since 2011 and the presence of competent Culicoides vector species throughout China. PPRV continues to circulate in western and northwestern China. SPPV and GTPV are endemic across major sheep-producing provinces. *Brucella* spp., particularly *B. melitensis*, remains one of the most important zoonotic pathogens in pastoral regions. *T. gondii* and *C. abortus* are leading infectious causes of ovine abortion, while BDV and CAEV cause chronic, insidious infections that are frequently underdiagnosed.

The assay was validated for sensitivity, specificity, precision, and clinical performance using reference materials and field clinical samples. The results demonstrate that the Panel 1/Panel 2 multiplex assay provides a reliable, high-throughput tool for the surveillance and differential diagnosis of major ovine infectious diseases.

## 2. Materials and Methods

### 2.1. Target Pathogens and Primer-Probe Design

The Panel 1 assay targets five ovine viral pathogens: BTV (NS3 gene), FMDV (3D gene), SBV (N gene), PPRV (M gene), and SPPV-GTPV (GPCR gene). The Panel 2 assay targets five bacterial/parasitic pathogens: BDV (UTR gene), Bru (IS711 gene), *T. gondii* (B1 gene), *C. abortus* (ompA gene), and CAEV (gag gene). All primer and probe sequences were designed based on conserved regions of the respective target genes and synthesized commercially (Shanghai Sangon Biotech, Shanghai, China). Each probe was labeled with a distinct fluorophore (Table 1).

### 2.2. Nucleic Acid Extraction

Total nucleic acids (including both DNA and RNA) were simultaneously extracted from 200 μL of blood, nasal swab, fecal swab, or tissue homogenate using a magnetic bead-based viral/bacterial nucleic acid extraction kit (Tianlong, Xi’an, China). All extraction procedures followed the manufacturer’s instructions. This kit enables co-extraction of DNA and RNA in a single workflow. An exogenous extraction internal control (Internal Control (IC)) was spiked into each sample prior to extraction to monitor extraction efficiency and the potential presence of PCR inhibitors. The extracted nucleic acids were eluted in 80 μL of RNase-free water and stored at −20 °C until use. All extraction procedures were performed in a biosafety cabinet, with strictly separated pre-amplification and post-amplification areas to prevent cross-contamination.

### 2.3. Pentaplex Real-Time Fluorescent PCR System Optimization

The pentaplex real-time fluorescent PCR system was established by optimizing the concentrations of primers and probes for each target. Primers and probes were diluted 10-fold serially from stock solutions and mixed to prepare the primer-probe mixture. The final reaction volume was 25 μL, containing 12.5 μL of One step PrimeScript III RT-qPCR Mix (TaKaRa, Shiga, Japan), 1.5 μL of primer-probe mixture, 5 μL of template, and 6 μL of nuclease-free water. The reactions were run on a JLM QX600 real-time fluorescent PCR instrument (Jielaimi, Chengdu, China) with the following thermocycling conditions: 50 °C for 5 min, 95 °C for 20 s; 40 cycles of 95 °C for 10 s and 60 °C for 30 s (fluorescence signal acquired). Each run included a positive control (a mixture of quantified plasmids at known concentrations) and a no-template control (NTC).

The internal control used the HEX fluorescent channel. Samples with invalid internal control signals (no amplification in the HEX channel) were excluded from analysis. Prior to formal testing, singleplex and multiplex standard curves were compared to verify that the amplification efficiency of the multiplex system was not compromised. All targets showed R^2^ values greater than 0.99, confirming no significant competitive inhibition among primers and probes in the multiplex format.

### 2.4. Digital PCR Quantification of Plasmids and Standard Curve

Plasmids containing target gene fragments were linearized with QuickCut Sac I and QuickCut Kpn I (TaKaRa, Shiga, Japan) at 37 °C for 15 min, then diluted to 10^3^ copies/μL. The exact copy number was determined using a JLM Digital Matrix-5000 digital PCR system (Jielaimi, Chengdu, China). A 10-fold serial dilution series (10^6^ to 10^1^ copies/μL) of the quantified plasmids was prepared for standard curve generation. Both singleplex and multiplex standard curves were constructed by plotting the cycle threshold (Ct) value against the logarithm of the plasmid copy number to verify whether the multiplex real-time PCR panel can work well.

### 2.5. Comparison of Singleplex and Multiplex Real-Time Fluorescent PCR System

To validate the interference of the multiplex reaction system on amplification efficiency and sensitivity, the above 10 plasmids were normalized to equal concentrations, pooled, and serially diluted 10-fold. Each dilution was subjected to both singleplex and multiplex real-time PCR, and the resulting Ct values were compared to assess potential interference of the multiplex format on assay performance.

### 2.6. Validation of Pathogen Specimen

To validate the detection performance of the panels established in this study on the target pathogens, a total of 111 known positive specimens were tested, covering seven pathogens—FMDV (types O, A, and Asia 1), PPRV, SPPV/GTPV, *Brucella* spp., *T. gondii*, *C. abortus*, and CAEV—obtained from vaccine strains or isolated from infected animals. Following the WOAH-recommended real-time PCR/RT-PCR or conventional PCR protocols, the fluorescent PCR assay was validated using seven reference pathogens under the same nucleic acid detection conditions as those employed for the standard plasmid testing, with detection performance assessed by Ct values.

### 2.7. Analytical Sensitivity and Limit of Detection at 95% Probability (LOD95)

The 95% limit of detection (LOD95) was calculated using Probit regression analysis. For each target, obtained from quantified plasmid-derived and pathogen-derived samples, the serial dilutions were tested with at least 8 replicates per concentration. Positive detection rates at each dilution level were fitted to a Probit regression model against log10-transformed template copy number [19]. The LOD95 was defined as the concentration at which the probability of detection reached 95%, calculated as LOD95 = 10^[(1.6449 − beta0)/beta1], where beta0 and beta1 are the intercept and slope of the fitted Probit model, and 1.6449 is the Probit value corresponding to the 95th percentile of the standard normal distribution. Data analysis was performed using WPS v12.1.0.28043 software.

### 2.8. Specificity Testing

Cross-reactivity was assessed using high-concentration plasmids (10^6^ copies/μL) of non-target pathogens, including plasmids from all targets within the same panel and selected plasmids from the opposite panel (e.g., BDV, Bru, *T. gondii*, *C. abortus*, and CAEV plasmids for Panel 1 testing; BTV, FMDV, SBV, PPRV, and SPPV-GTPV plasmids for Panel 2 testing). Additionally, ovine genomic DNA was also evaluated as a target for probe cross-reactivity assessment.

### 2.9. Testing of Precision and Reproducibility

Precision was evaluated at two template concentrations (10^5^ and 10^3^ copies/μL) using equimolar mixtures of quantified plasmids. For intra-assay precision, six replicates were tested in a single run. For inter-assay precision, the same panel was tested once daily for five consecutive days. The coefficient of variation (CV) of Ct values was calculated for each target channel.

### 2.10. Clinical Sample Testing and Concordance Analysis

A total of 620 clinical samples were collected from Ningxia, Lanzhou, Gansu, and other regions of China. These samples included 240 nasal swabs, 260 fecal swabs, and 120 tissue samples. All samples were tested for rapid screening of multiple pathogens using the panel 1/panel 2 multiplex assay. The positive results were then confirmed by WOAH-recommended PCR or real-time PCR for each target to assess the concordance between the multiplex panel and the standard methods.

Although classified as respiratory or reproductive pathogens, these ten agents can be sampled via nasal or fecal swabs owing to their shedding routes. Respiratory viruses (e.g., BTV, PPRV) are shed via both respiratory and oral/fecal routes, whereas reproductive pathogens (e.g., *Brucella* spp., *C. abortus*) may be excreted in feces during active infection or abortion. This strategy aligns with international trade quarantine standards, and pooled specimen types increase detection likelihood across diverse shedding patterns.

### 2.11. Statistical Analysis

Data analysis was performed using WPS v12.1.0.28043. Standard curves were generated by linear regression (Ct vs. log_10_ copy number). R^2^, slope, and amplification efficiency (E) were calculated. Ct values were compared using Student’s *t*-test, with *p* < 0.05 considered statistically significant.

### 2.12. Ethics Statement

All animal experiments were performed in compliance with the Guidelines on the Humane Treatment of Laboratory Animals (Ministry of Science and Technology of the People’s Republic of China, Policy No. 2006398) and were approved by the Institutional Animal Care and Use Committee at the Shanghai Veterinary Research Institute (IACUC No: Shvri-Pi-0124).

## 3. Results

### 3.1. Standard Curve and Amplification Efficiency

**Figure 1:**
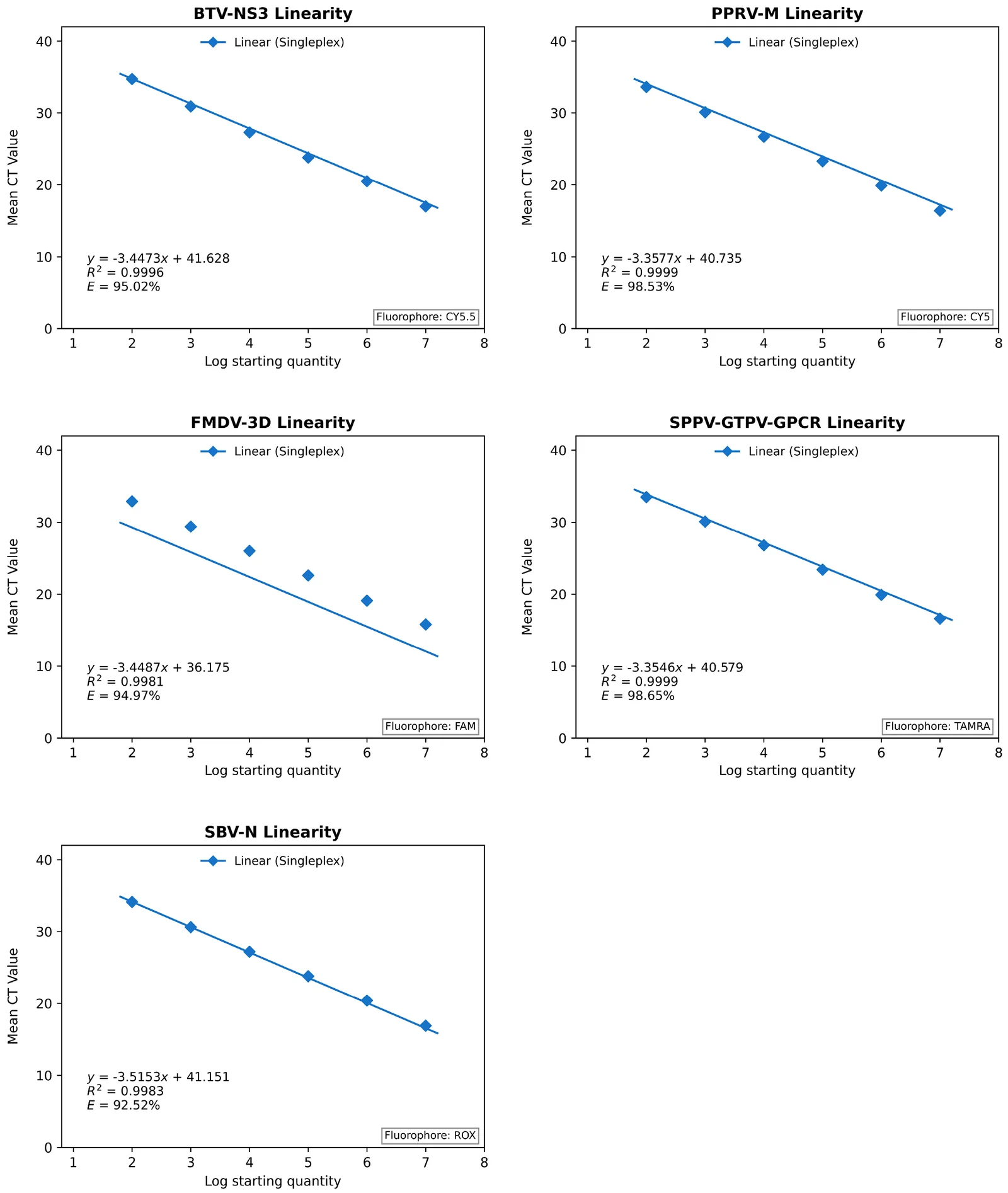
Standard curves for Panel 1 (respiratory pathogens) in singleplex real-time fluorescent PCR format. Linear regression plots of Ct values versus log10 plasmid copy number for BTV (CY5.5), FMDV (FAM), SBV (ROX), PPRV (CY5), and SPPV-GTPV (TAMRA). All targets demonstrated high linearity with R^2^ ranging from 0.9981 to 0.9999 and amplification efficiency between 92% and 98%.

**Figure 2:**
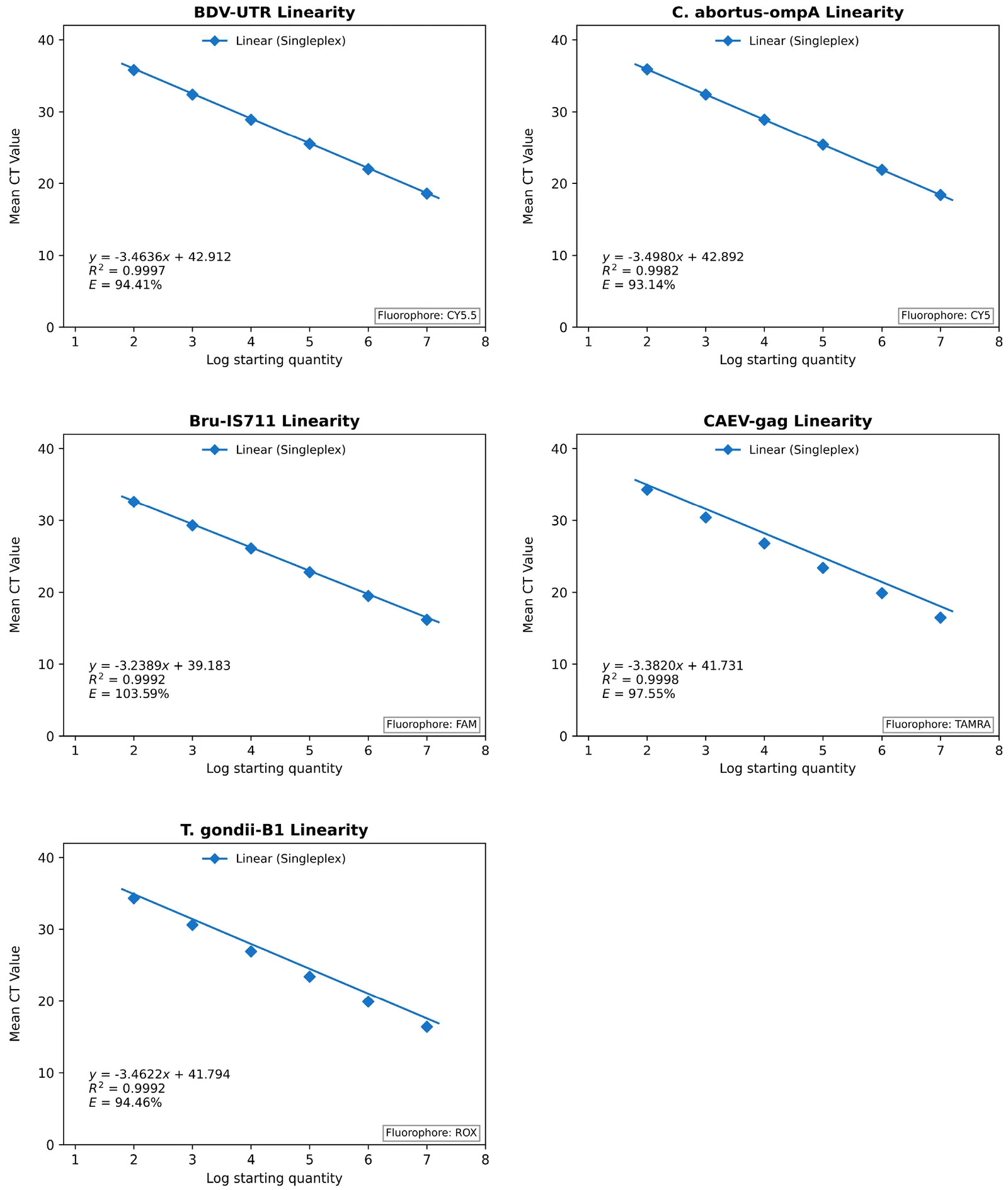
Standard curves for Panel 2 (reproductive pathogens) in singleplex real-time fluorescent PCR format. Linear regression plots for BDV (CY5.5), CAEV (TAMRA), Bru (FAM), *C. abortus* (CY5), and *T. gondii* (ROX). All curves demonstrated high linearity (R^2^ ranging from 0.9982 to 0.9998) and consistent amplification efficiency across the dynamic range.

**Figure 3:**
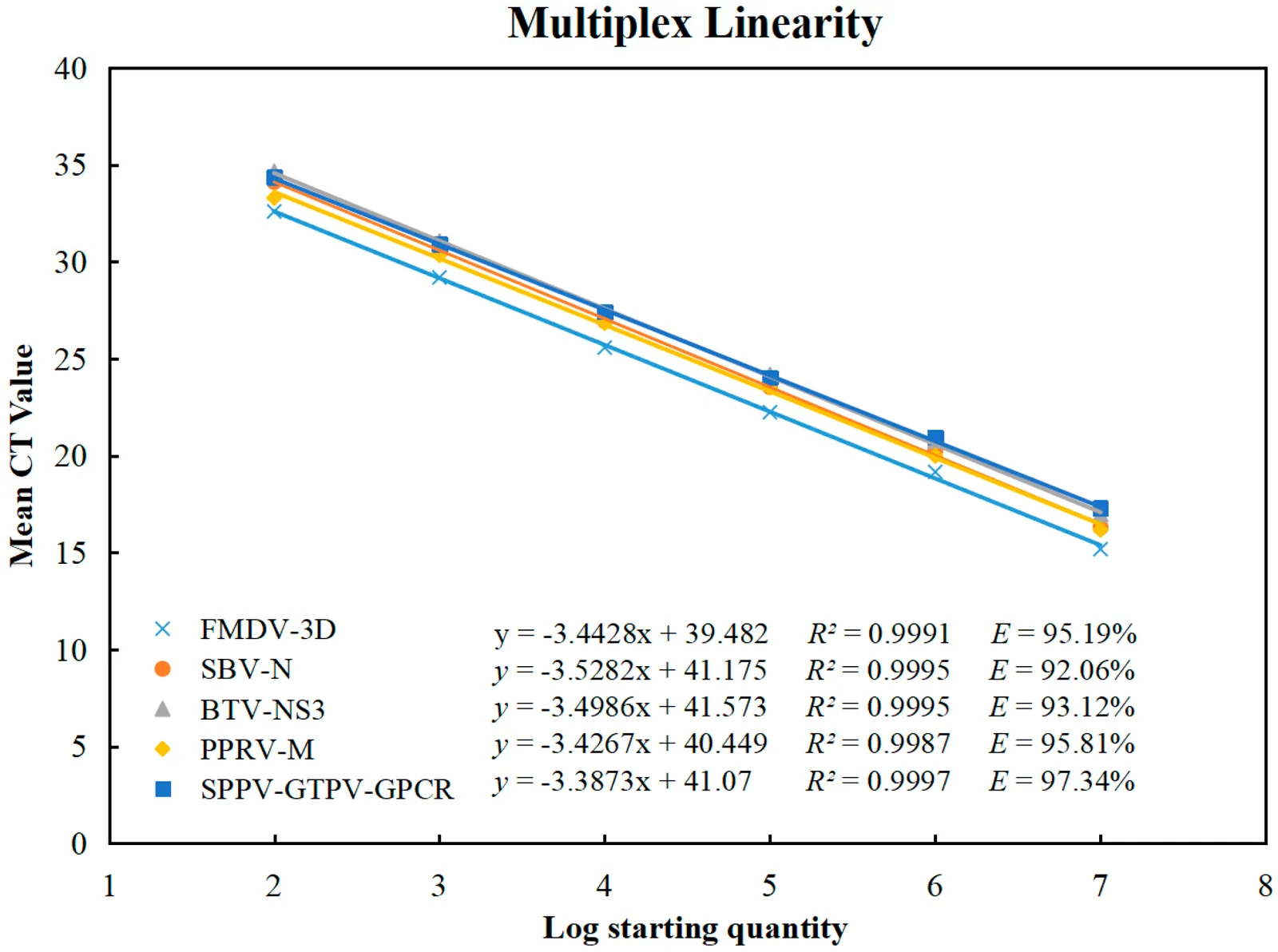
Standard curves for Panel 1 (respiratory pathogens) in multiplex real-time fluorescent PCR format. Linear regression plots of Ct values versus log10 plasmid copy number for BTV (CY5.5), FMDV (FAM), SBV (ROX), PPRV (CY5), and SPPV-GTPV (TAMRA). All targets exhibited robust linearity, with R^2^ ranging from 0.9987 to 0.9997 and amplification efficiency between 92% and 97% in multiplex format, and no cross-reactivity was observed between channels.

**Figure 4:**
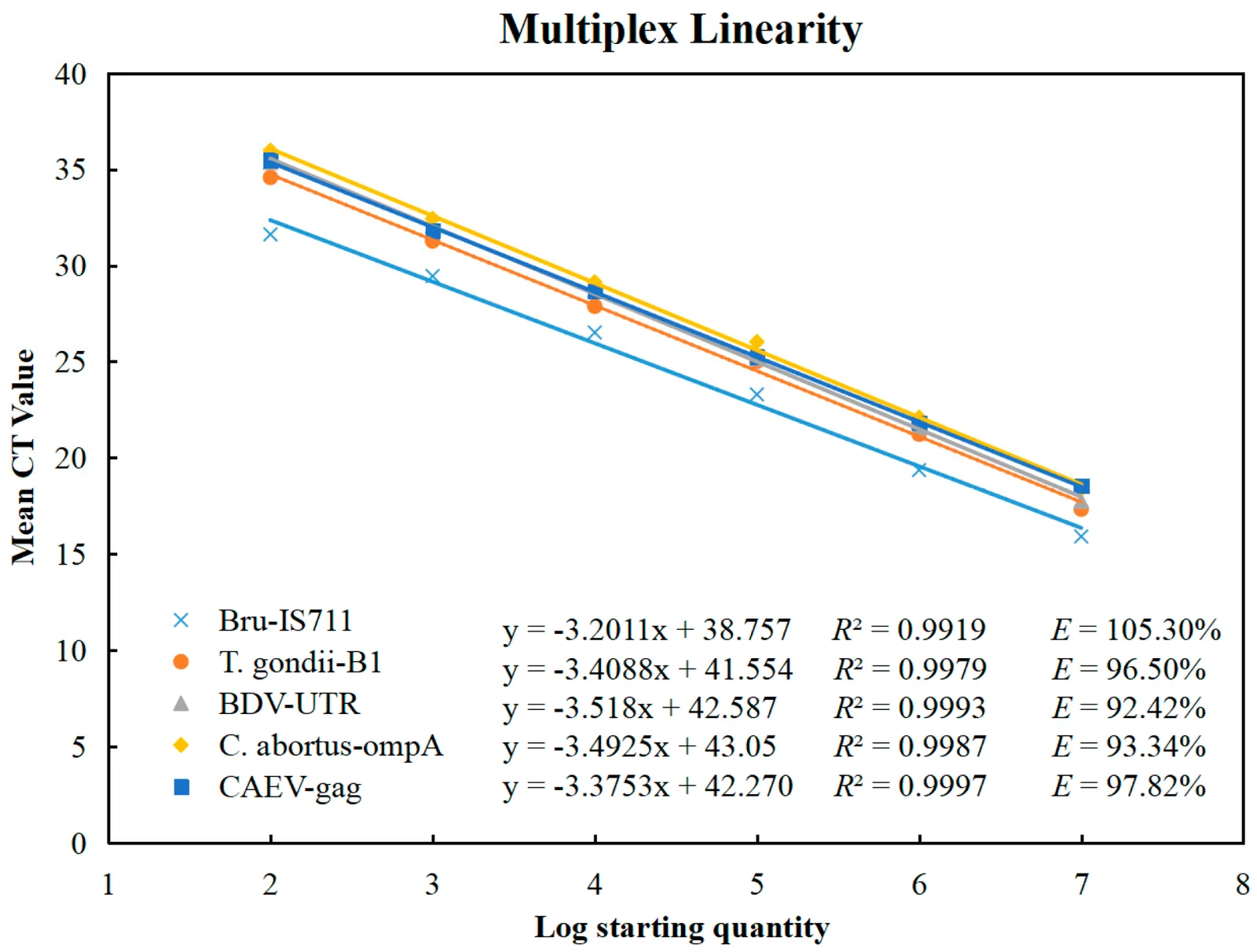
Standard curves for Panel 2 (reproductive pathogens) in multiplex real-time fluorescent PCR format. Linear regression plots for BDV (CY5.5), CAEV (TAMRA), Bru (FAM), *C. abortus* (CY5), and *T. gondii* (ROX). All targets displayed consistent linearity, with R^2^ ranging from 0.9919 to 0.9997 and amplification efficiency between 92% and 105% in multiplex format, and no cross-reactivity was observed between channels.

The plasmids were linearized, diluted, and precisely quantified using digital PCR. Copy numbers were determined as follows: BTV, 645 copies/μL; FMDV, 556 copies/μL; SBV, 1022 copies/μL; PPRV, 531 copies/μL; SPPV-GTPV, 213 copies/μL; BDV, 177 copies/μL; Bru, 834 copies/μL; *T. gondii*, 2089 copies/μL; *C. abortus*, 329 copies/μL; and CAEV, 382 copies/μL (Table 2). Standard curves were generated for all ten targets in both singleplex and multiplex formats. All standard curves obtained from singleplex and multiplex real-time PCR were identical, showing strong linearity with R^2^ values ranging from 0.9981 to 0.9999 and 0.9987 to 0.9997 (Panel 1 in singleplex and multiplex format, respectively) and 0.9982 to 0.9998 and 0.9919 to 0.9997 (Panel 2 in singleplex and multiplex format, respectively) (Figure 1, Figure 2, Figure 3 and Figure 4). The amplification efficiencies are more than 92%, and no cross-reactivity is observed between the channels, confirming that the developed multiplex real-time PCR performance is robust, and the mixtures of all the primers and probes can work well together in multiplex format.

### 3.2. Comparison of Singleplex and Multiplex Real-Time Fluorescent PCR

**Figure 5:**
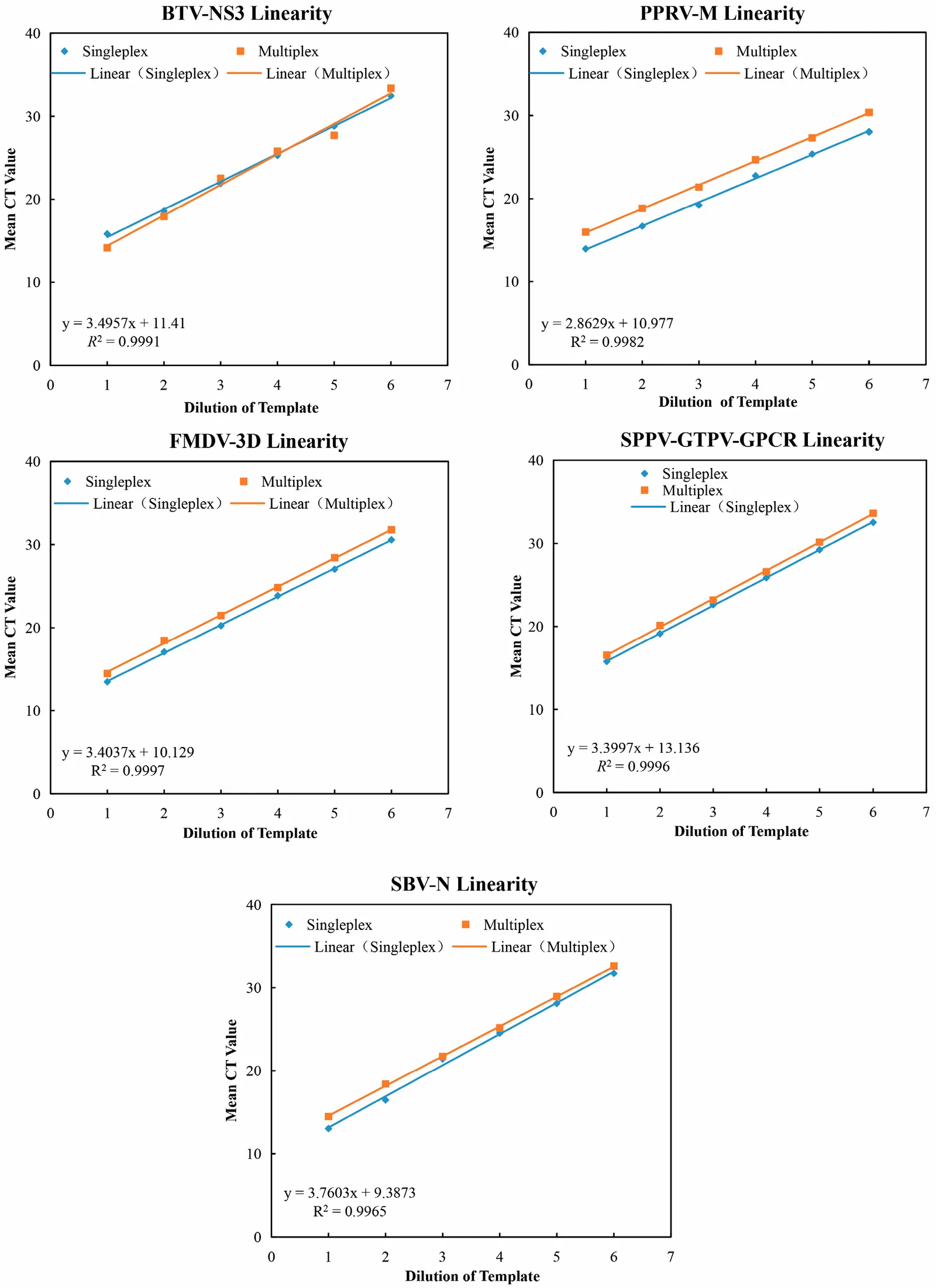
Comparison of singleplex and multiplex real-time fluorescent PCR for Panel 1. Regression analysis showed high coefficiency with R^2^ ranging from 0.9965~0.9997 among all the targets between the singleplex and multiplex systems. Ct values were comparable between singleplex and multiplex reactions at the same template concentrations, with no significant interference among all targets in the multiplex system.

**Figure 6:**
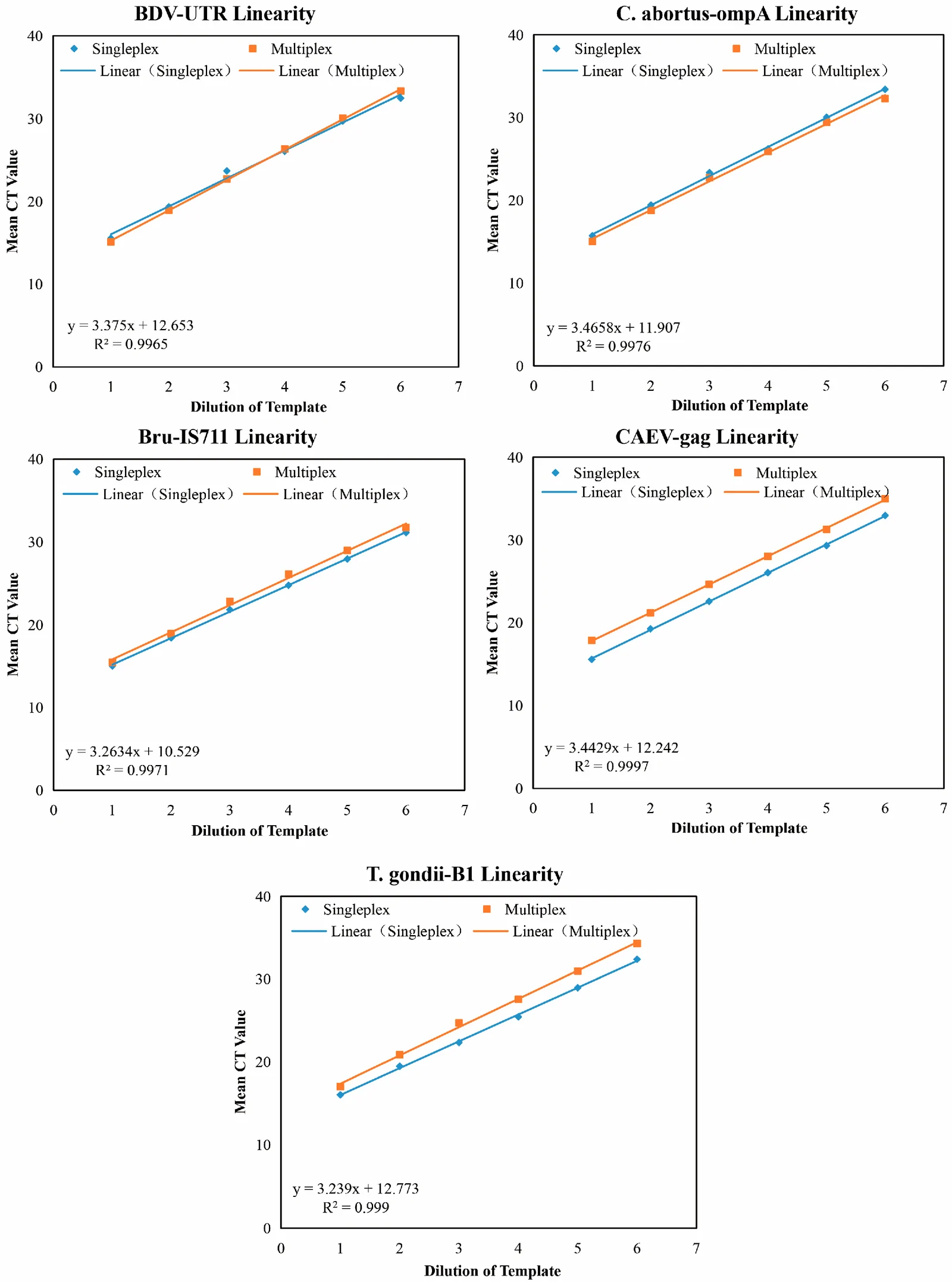
Comparison of singleplex and multiplex real-time fluorescent PCR for Panel 2. Regression analysis showed high coefficiency with R^2^ ranging from 0.9965 to 0.9997 among all the targets between the singleplex and multiplex systems. Ct values were comparable between singleplex and multiplex reactions at the same template concentrations, with no significant interference among all targets in the multiplex system.

Using different dilutions of the same mixed template, singleplex and multiplex real-time fluorescent PCR were performed separately to compare the consistency between singleplex and multiplex amplifications. Regression analysis revealed that the coefficient of determination (R^2^) ranged from 0.9965 to 0.9997 for all targets between singleplex and multiplex real-time fluorescent PCR, indicating a strong consistency between them (Figure 5 and Figure 6). For templates at the same concentration, the Ct values obtained from singleplex and multiplex amplifications were essentially comparable with good linearity, and no marked interference was observed among all targets in the multiplex amplification system.

### 3.3. Testing Results of Pathogen Specimen Validation

A total of 111 known positive specimens covering seven pathogens—FMDV (types O, A, and Asia 1), PPRV, SPPV/GTPV, *Brucella* spp., *T. gondii*, *C. abortus*, and CAEV—were tested using the panels developed in this study, with WOAH-recommended PCR protocols serving as the reference methods. Of the 111 specimens, 109 were correctly identified, yielding an overall concordance of 98.2% (109/111). All vaccine-derived specimens (*n* = 17; FMDV types O, A, and Asia 1, PPRV, SPPV/GTPV, *Brucella* spp., and *C. abortus*) were detected with 100% concordance, with Ct values ranging from 14.89 to 20.20. For specimens isolated from infected animals (*n* = 94), the concordance was 97.9% (92/94), with Ct values ranging from 19.19 to 36.46. Two specimens—one *Brucella* spp. and one *C. abortus*—were not detected by the developed panels, corresponding to individual concordance rates of 95.65% (22/23) and 93.3% (14/15), respectively. These results demonstrate that the method can effectively detect the target pathogens (Table 3).

### 3.4. Analytical Sensitivity and LOD

The LOD95 was determined by testing serial dilutions of quantified plasmids. In the singleplex format, all ten targets achieved an LOD95 ranging from 2.3 to 5.6 copies/μL (Table 4). In the multiplex format, the LOD95 ranged from 2.9 to 6.1 copies/μL for all targets, although the Ct values were slightly higher than those in the singleplex format (difference < 1 Ct), which is consistent with typical multiplex real-time fluorescent PCR behavior. These results demonstrate that the multiplex assay maintains high analytical sensitivity suitable for clinical diagnostic applications.

To further validate the pathogen-derived LOD95, genomic nucleic acids from reference strains of two representative pathogens—BTV (RNA virus) and *T. gondii* (DNA pathogen)—were extracted, quantified by spectrophotometry, and tested using the singleplex and multiplex assay. The pathogen-derived LOD values for BTV (4.4 and 5.2 copies/μL) and *T. gondii* (4.3 and 5.8 copies/μL) were slightly higher than but consistent with the corresponding plasmid-derived values (Table 4), confirming that plasmid standards provide a reliable surrogate for LOD95 evaluation in this multiplex system.

### 3.5. Specificity

No cross-reactivity was observed between any of the target pairs within the same multiplex panel. In the Panel 1 assay (detecting BTV, FMDV, SBV, PPRV, and SPPV-GTPV), none of the plasmids from Panel 2 targets (BDV, Bru, *T. gondii*, *C. abortus*, and CAEV) or additional plasmids (LSDV-ORF101, LSDV-ORF126) produced false-positive signals. Similarly, in the Panel 2 assay, no cross-reactivity was detected among Panel 1 targets or with additional tested plasmids. The NTC reactions showed no amplification in any channel, confirming the absence of reagent contamination. Melting curve analysis confirmed the identity of the amplified products (Table 5). Additionally, ovine genomic DNA tested negative in all channels, confirming the absence of host DNA interference. (Table 5).

### 3.6. Results of Precision and Reproducibility

The results of intra-assay and inter-assay precision testing are summarized in Table 5 and Table 6. At both high (10^5^ copies/μL) and low (10^3^ copies/μL) template concentrations, the intra-assay CV was less than 1% for all targets in both Panel 1 and Panel 2 assays. The inter-assay CV was less than 1.5% for all targets in the Panel 1 assay and less than 1% in the Panel 2 assay. All values met the performance criterion of CV less than 5%, demonstrating superior repeatability and reproducibility of the multiplex assay (Table 6 and Table 7).

### 3.7. Clinical Sample Testing

A total of 620 clinical samples (240 nasal swabs, 260 fecal swabs, and 120 tissue samples) collected from Ningxia, Lanzhou, Gansu, and other regions of China were tested using the panel 1/panel 2 multiplex assay. The testing results for clinical samples are shown in Table 8. In the panel 1 assay, BTV was detected in 4 samples, FMDV in 13 samples, SBV in 0 samples, PPRV in 30 samples, and SPPV-GTPV in 0 samples. In the panel 2 assay, BDV was detected in 0 samples, Bru in 69 samples, *T. gondii* in 25 samples, *C. abortus* in 43 samples, and CAEV in 16 samples. The confirmation results of screened positives by WOAH-recommended methods were largely consistent with screening results, except for PPRV in panel 1 with 93.33% (28/30) and *T. gondii* in panel 2 with 92% (23/25) (Table 8). The minor discrepancies are expected because the real-time PCR screening panel has higher sensitivity than conventional PCR recommended by WOAH for PPRV and *T. gondii*, as low-copy samples may be missed by the less sensitive conventional PCR. The positive percent agreement (Positive Percent Agreement (PPA)) was 98% (196/200), and negative percent agreement (Negative Percent Agreement (NPA)) was 99% (420/424), indicating strong concordance between the multiplex assay and WOAH standard methods, and the multiplex panels are a good alternative for rapid screening applications.

## 4. Discussion

In this study, we successfully developed and validated a pentaplex real-time fluorescent PCR assay (Panel 1/Panel 2) for simultaneous detection of ten major ovine pathogens, including five respiratory pathogens and five reproductive pathogens [17]. The assay demonstrated outstanding analytical performance, including high sensitivity (LOD95 < 6.1 copies/μL), high specificity (no cross-reactivity), and superior precision (CV < 1.5%), making it suitable for routine diagnostic applications and large-scale surveillance programs.

The selection of target genes was based on publicly available genomic sequences and conservation across geographic strains. For BTV, the NS3 gene was selected as it encodes a relatively conserved structural protein [20]. The 3D gene of FMDV, which encodes the RNA-dependent RNA polymerase, is highly conserved and widely used in FMDV diagnostics. The N gene of SBV and the M gene of PPRV are also well-conserved and have been validated in previous studies. The GPCR gene of SPPV-GTPV, encoding a viral envelope glycoprotein, provides a specific target for SPPV-GTPV detection [21,22,23,24]. For Panel 2 targets, the UTR gene of BDV was selected as it contains the most conserved genomic region among pestiviruses, providing a robust basis for BDV detection with high sensitivity and specificity. The IS711 insertion sequence, a Brucella-specific genetic element present in multiple copies in the genome, serves as an ideal target for *Brucella* spp. identification and has been widely adopted in molecular diagnostics. The B1 gene of *T. gondii*, encoding a tachyzoite-specific protein, is one of the most frequently used targets in *T. gondii* PCR detection due to its high copy number and species specificity. The ompA gene of *C. abortus*, encoding a highly conserved outer membrane protein, offers a reliable and specific target for *C. abortus* detection in clinical samples. The gag gene of CAEV, encoding the group-specific antigen polyprotein, is a well-established target for CAEV diagnostics and has demonstrated consistent detection performance across diverse viral strains [25,26,27,28].

One of the key challenges in multiplex real-time fluorescent PCR is maintaining equal sensitivity for all targets, especially when targets are present at very different concentrations [17]. In this study, we optimized the primer and probe concentrations to minimize competition between primer pairs and ensure balanced amplification. The comparison of singleplex and multiplex standard curves confirmed that the amplification efficiency was not compromised in the multiplex format (R^2^ > 0.99 for all targets). The slight increase in Ct values in the multiplex format compared to singleplex is within the expected range and does not affect the clinical sensitivity.

Clinical validation in this study was limited to seven of the ten target pathogens due to the unavailability of well-characterized positive specimens for the remaining three targets (BTV, SBV, and BDV). All ten pathogens were successfully validated at the plasmid level through standard curve construction and analytical performance assessment (Figure 1, Figure 2, Figure 3 and Figure 4). Specimen-level validation for BTV, SBV, and BDV will be conducted in future studies as appropriate clinical samples become available. Collectively, the high concordance between the developed panels and the WOAH-recommended reference methods across the seven validated pathogens and 200 screening-positive samples of 620 clinical samples (Table 8) supports the applicability of this multiplex real-time fluorescent PCR assay for the rapid screening and simultaneous differentiation of the target ovine pathogens in clinical settings.

The internal control (IC) strategy is critical for multiplex assays to distinguish between true negatives and false negatives caused by extraction failure or inhibition. We incorporated an exogenous IC (HEX channel) into each reaction to monitor the entire testing process, from nucleic acid extraction to amplification. Samples with negative IC signals were flagged as invalid and retested, ensuring the reliability of the results.

Clinical validation using 620 field samples collected from multiple provinces demonstrated the practical utility of the assay. The strong concordance with reference methods (PPA and NPA both >98%) confirms that the Panel 1/Panel 2 multiplex assay is suitable for use in real-world diagnostic settings [29]. The less-than-perfect concordance observed for these two pathogens is most plausibly attributable to the inherent complexity of clinical specimens. Multiple factors—including low pathogen load, PCR inhibitors present in the sample matrix, and nucleic acid degradation during prolonged storage—can independently or synergistically compromise real-time PCR performance. Retrospective review of the discrepant cases suggests that these non-concordant results likely reflect the convergence of multiple adverse factors within individual samples. Although singleplex real-time PCR exhibits robust performance under such conditions, multiplex formats may demonstrate marginally reduced detection sensitivity when confronted with highly complex samples, particularly when multiple compounding variables coexist. Nevertheless, the overall concordance rates achieved by our multiplex assay across 620 clinical samples confirm that the system maintains satisfactory analytical robustness and diagnostic reliability even when applied to specimens of considerable complexity.

The ability to simultaneously detect ten pathogens in two reactions greatly improves throughput and reduces the cost per test, which is particularly valuable for resource-limited laboratories and large-scale screening programs. From a translational perspective, the pentaplex-panel format offers several practical advantages. Total turnaround time from extraction to result is approximately 2.5 h. The integration of ten pathogens into two pentaplex reactions reduces reagent consumption by approximately two-thirds compared to ten individual PCRs, making this approach highly cost-effective for large-scale surveillance. The assay is well-suited for: (i) port quarantine screening of imported sheep and goats; (ii) international trade certification; (iii) endemic disease surveillance in major sheep-producing regions; and (iv) outbreak investigation requiring rapid differential diagnosis.

This study offers several innovative contributions. First, this is the first multiplex assay to systematically design primer-probe sets targeting ten major ovine pathogens. Second, through systematic combinatorial optimization, we successfully integrated five respiratory and five reproductive pathogens into two balanced pentaplex reactions (Panel 1 and Panel 2). The separation into two syndrome-based panels allows clinicians to order only the relevant panel based on clinical presentation. Third, this study is particularly relevant to increasing international trade in live animals. The rapid, accurate, multi-pathogen detection capability holds significant economic value for customs inspection and port quarantine, where simultaneous screening of multiple transboundary animal diseases can substantially reduce clearance time.

Compared with existing singleplex or multiplex real-time fluorescent PCR methods for ovine pathogens (Table 9), the present assay offers a broader target spectrum (ten pathogens spanning RNA viruses, DNA viruses, bacteria, and a parasite) and a syndrome-based panel design (respiratory vs. reproductive) that optimizes clinical workflow. Although several multiplex PCR assays have been reported, the overlap of target pathogens with the present study is remarkably limited (10–50%). This reflects distinct clinical focuses: previous studies primarily targeted abortive agents or broad-spectrum pathogens, whereas the present assay specifically focuses on ovine respiratory and reproductive pathogens of international trade importance. All 10 pathogens targeted in this assay are WOAH-listed notifiable diseases critical for import/export quarantine.

Several limitations should be acknowledged. First, cross-reactivity testing against closely related pathogens was not fully exhaustive due to limited reference strain availability. EHDV has not been reported in China, and reference materials were unobtainable. Cross-reactivity with other morbilliviruses could not be assessed. For *Brucella* spp., differentiation among species was not investigated. Second, although plasmid-based validation confirmed analytical performance, validation using inactivated whole-pathogen reference strains would further strengthen clinical applicability, as recommended by the WOAH Terrestrial Manual. Third, the clinical sample size was limited to 620 samples from three regions; larger multicenter studies are warranted. In future studies, we will continue to perform nucleic acid sample analysis for all ten pathogens.

## 5. Conclusions

The two pentaplex real-time fluorescent PCR assays developed in this study offer a fast, highly sensitive, specific, and dependable tool for the rapid screening and differential detection of major ovine respiratory and reproductive pathogens. The assay can be readily implemented in veterinary diagnostic laboratories and contributes to improved disease surveillance and control strategies for small ruminants.

## Figures and Tables

**Table 1:** Primer and Probe Sequences and Fluorophore Labeling.

Target Pathogen	Gene	Primer/Probe	Sequence (5′→3′)	Fluorophore	Usage	Genebank ID
BTV	NS3	BTV-F	GTTGCCCTTGAAATYTTGGACAA	—	panel 1	PX861143.1
BTV	NS3	BTV-R	CTYAAYCTTACATCATCACGAAAC	—	panel 1	
BTV	NS3	BTV-P	ATGTCAAAYACAACTGGTGCAACGCAA	CY5.5	panel 1	
FMDV	3D	FMDV-F	GGAACTGGGTTTTACAAACCTGT	—	panel 1	MT495499.1
FMDV	3D	FMDV-R	GAGTCCTGCCACGGAGATC	—	panel 1	
FMDV	3D	FMDV-P	CTCTCCTTTGCACGCCGTGGG	FAM	panel 1	
SBV	N	SBV-F	AACAACACATGYGACACCAA	—	panel 1	NC_043582.1
SBV	N	SBV-R	GGCACTTCCACCTCATCAAC	—	panel 1	
SBV	N	SBV-P	CGACACGGCCTTCCGGCTG	ROX	panel 1	
PPRV	M	PPRV-F	GGTGGAACAAGTCTCC	—	panel 1	OR419686.1
PPRV	M	PPRV-R	GCCCGCCAGAGATATCGG	—	panel 1	
PPRV	M	PPRV-P	GAACCCCAGCTGTGCATGG	CY5	panel 1	
SPPV-GTPV	GPCR	SPPV-GTPV-F	GGTGTTTCCTTTTAATTTATAC	—	panel 1	KF661979.1
SPPV-GTPV	GPCR	SPPV-GTPV-R	ACTGGGTAAACTATAGCTAGG	—	panel 1	
SPPV-GTPV	GPCR	SPPV-GTPV-P	CCCAAACTCCATTGTTTAGCTATAC	TAMRA	panel 1	
BDV	UTR	BDV-F	GAGATGCCACGTGGACGAG	—	panel 2	GQ902940.1
BDV	UTR	BDV-R	CAACTCCATGTGCCATGTACAGC	—	panel 2	
BDV	UTR	BDV-P	GTGGGCCTCTGCAGCACC	CY5.5	panel 2	
CAEV	gag	CAEV-F	GCAACTGCAAACAGTAGC	—	panel 2	PP382771.1
CAEV	gag	CAEV-R	GGCATCATGGCCAATAC	—	panel 2	
CAEV	gag	CAEV-P	GCAGCATGGCCTCGTGTC	TAMRA	panel 2	
Bru	IS711	Bru-F	GCCGGATAGAAGGCTTGAAGC	—	panel 2	CP069385.1
Bru	IS711	Bru-R	CGCGCGGTGGATTGACGACC	—	panel 2	
Bru	IS711	Bru-P	GGGTGTTGGCTTTATTCGCAGCG	FAM	panel 2	
*C. abortus*	ompA	*C. abortus*-F	GGGAAGGTGCTTCAGGTGATCC	—	panel 2	LC554223.1
*C. abortus*	ompA	*C. abortus*-R	CGTAGTATCCTGCGCGGATGC	—	panel 2	
*C. abortus*	ompA	*C. abortus*-P	GCGATCCTTGCTCTACTTGG	CY5	panel 2	
*T. gondii*	B1	*T. gondii*-F	GCATAGGTTGCAGTCAC	—	panel 2	MZ717192.1
*T. gondii*	B1	*T. gondii*-R	GGTCAACTATCGATTGCAGGCG	—	panel 2	
*T. gondii*	B1	*T. gondii*-P	CCTCTGCTGGCGAAAAGTGAAATTC	ROX	panel 2	
Internal Control	mt	IC-F	ACCGTGCAAAGGTAGCATAATCA	—	Both	OR459762.1
Internal Control	mt	IC-R	GCTCCATAGGGTCTTCTCGTCTT	—	Both	
Internal Control	mt	IC-P	ACTGTCTCTTACTTCCAATCAGTGA	HEX	Both	

F: forward, R: reverse, P: Probe.

**Table 2:** Plasmid Quantification by Digital PCR.

Assay	Target	Plasmid ID	Digital PCR (Copies/μL)
Panel 1	BTV	Panel 1-BTV-NS3	645
Panel 1	FMDV	Panel 1-FMDV-3D	556
Panel 1	SBV	Panel 1-SBV-N	1022
Panel 1	PPRV	Panel 1-PPRV-M	531
Panel 1	SPPV-GTPV	Panel 1-SPPV-GTPV-GPCR	213
Panel 2	BDV	Panel 2-BDV-UTR	177
Panel 2	Bru	Panel 2-Bru-IS711	834
Panel 2	*T. gondii*	Panel 2-*T. gondii*-B1	2089
Panel 2	*C. abortus*	Panel 2-*C. abortus*-ompA	329
Panel 2	CAEV	Panel 2-CAEV-gag	382

**Table 3:** Validation of pathogens.

Target Pathogen	Subtype/Source	Origin	Reference Test	Results (Panels Developed in This Study)	Consistency
FMDV	5 O-FMDV positive samples	OAK/93 strain, DR/80 strain, OS/99 strain, Re-O/Ind-2001/2003 strain and OMH/02 strain for inactivated vaccines	Real-time RT-PCR of WOAH terrestrial manuals	5(+)/Ct = 15.23~18.14	100% (5/5)
	3 A-FMDV positive samples	AF/72 strain, Re-A/WH/09 strain and AKT-III strain for inactivated vaccines	Real-time RT-PCR of WOAH terrestrial manuals	3(+)/Ct = 14.89~17.25	100% (3/3)
	2 Asia1-FMDV positive samples	Asia1 KZ/03 strain and Asia1/XJ/KLMY/04 strain for inactivated vaccines	Real-time RT-PCR of WOAH terrestrial manuals	2(+)/Ct = 16.12,16.78	100% (2/2)
	21 FMDV-positive samples	Infected animals	Real-time RT-PCR of WOAH terrestrial manuals	21(+)/Ct = 25.12~36.46	100% (21/21)
PPRV	1 PPRV positive sample	Clone 9 strain for attenuated live vaccines	RT-PCR of WOAH terrestrial manuals	1(+)/Ct = 18.39	100% (1/1)
	5 PPRV positive samples	Infected animals	RT-PCR of WOAH terrestrial manuals	5(+)/Ct = 22.37~32.90	100% (5/5)
SPPV/GTPV	1 SPPV-GTPV positive sample	CVCC AV41 Strains from attenuated live vaccines	Real-time PCR of WOAH terrestrial manuals	1(+)/Ct = 16.25	100% (1/1)
	8 SPPV-GTPV positive samples	Infected animals	Real-time PCR of WOAH terrestrial manuals	8(+)/Ct = 19.19~34.56	100% (8/8)
*Brucella* spp.	4 brucellosis-positive samples	Strain M5(CVCC-18), Strain M5-90, Strain S2, Strain Rev.1 from live vaccines	Real-time PCR of WOAH terrestrial manuals	4(+)/Ct = 16.96~20.18	100% (4/4)
	23 brucellosis-positive samples	Infected animals	Real-time PCR of WOAH terrestrial manuals	22(+)/Ct = 21.89~36.10 1(-)/no Ct	95.65 (22/23)
*T. gondii*	9 *T. gondii*	Infected animals	PCR of WOAH terrestrial manuals	9(+)/Ct = 21.84~33.33	100% (9/9)
*C. abortus*	1 *C. abortus* positive sample	*C. abortus* inactivated vaccine	Real-time PCR of WOAH terrestrial manuals	1(+)/Ct = 20.20	100% (1/1)
	15 *C. abortus* positive samples	Infected animals	Real-time PCR of WOAH terrestrial manuals	14(+)/Ct = 28.49~35.46 1(-)/no Ct	93.3% (14/15)
CAEV	13 CAEV-positive samples	Infected animals	Real-time PCR of WOAH terrestrial manuals	13(+)/Ct = 29.19~34.09	100% (13/13)

**Table 4:** Limit of Detection 95% (LOD95).

Assay	Target	Plasmid Singleplex LOD95 (Copies/μL)	Plasmid Multiplex LOD95 (Copies/μL)	Pathogen Singleplex LOD95 (Copies/μL)	Pathogen Multiplex LOD95 (Copies/μL)
Panel 1	BTV	3.7	4.1	4.4	5.2
Panel 1	FMDV	2.3	2.9	-	-
Panel 1	SBV	2.8	2.5	-	-
Panel 1	PPRV	3.1	3.9	-	-
Panel 1	SPPV-GTPV	4.8	5.4	-	-
Panel 2	BDV	3.1	3.6	-	-
Panel 2	CAEV	3.6	4.7	-	-
Panel 2	Bru	4.8	5.0	-	-
Panel 2	*T. gondii*	4.3	4.1	4.3	5.8
Panel 2	*C. abortus*	5.6	6.1	-	-

**Table 5:** Specificity (Cross-Reactivity) Testing Results.

Assay	Non-Target Plasmid Tested	Amplification in Target Channel	Conclusion
Panel 1	CAEV (Panel 2-CAEV-gag)	None	No cross-reactivity
Panel 1	Bru (Panel 2-Bru-IS711)	None	No cross-reactivity
Panel 1	*T. gondii* (Panel 2-*T. gondii*-B1)	None	No cross-reactivity
Panel 1	*C. abortus* (Panel 2-*C. abortus*-ompA)	None	No cross-reactivity
Panel 1	LSDV-ORF101	None	No cross-reactivity
Panel 1	LSDV-ORF126	None	No cross-reactivity
Panel 2	BTV (Panel 1-BTV-NS3)	None	No cross-reactivity
Panel 2	FMDV (Panel 1-FMDV-3D)	None	No cross-reactivity
Panel 2	SBV (Panel 1-SBV-N)	None	No cross-reactivity
Panel 2	PPRV (Panel 1-PPRV-M)	None	No cross-reactivity
Panel 2	SPPV-GTPV (Panel 1-SPPV-GTPV-GPCR)	None	No cross-reactivity
Both	NTC (no template)	None	No amplification
Both	Ovine gDNA	None	No amplification

**Table 6:** Intra-Assay Precision Results.

Assay	Target	10^5^ Copies/μL (Mean Ct ± SD)	10^5^ Copies/μL (CV%)	10^3^ Copies/μL (Mean Ct ± SD)	10^3^ Copies/μL (CV%)
Panel 1	BTV	16.23 ± 0.12	0.74%	24.15 ± 0.18	0.75%
Panel 1	FMDV	16.45 ± 0.11	0.67%	24.32 ± 0.15	0.62%
Panel 1	SBV	15.98 ± 0.10	0.63%	23.89 ± 0.14	0.59%
Panel 1	PPRV	16.67 ± 0.13	0.78%	24.51 ± 0.17	0.69%
Panel 1	SPPV-GTPV	17.12 ± 0.11	0.64%	24.89 ± 0.16	0.64%
Panel 2	BDV	16.34 ± 0.09	0.55%	24.28 ± 0.12	0.49%
Panel 2	CAEV	16.51 ± 0.08	0.48%	24.45 ± 0.11	0.45%
Panel 2	Bru	16.19 ± 0.07	0.43%	24.12 ± 0.10	0.41%
Panel 2	*T. gondii*	15.87 ± 0.06	0.38%	23.78 ± 0.09	0.38%
Panel 2	*C. abortus*	16.78 ± 0.08	0.48%	24.62 ± 0.10	0.41%

**Table 7:** Inter-Assay Precision Results (5 Days).

Assay	Target	10^5^ Copies/μL (Mean Ct ± SD)	10^5^ Copies/μL (CV%)	10^3^ Copies/μL (Mean Ct ± SD)	10^3^ Copies/μL (CV%)
Panel 1	BTV	16.31 ± 0.19	1.17%	24.22 ± 0.25	1.03%
Panel 1	FMDV	16.52 ± 0.22	1.33%	24.38 ± 0.28	1.15%
Panel 1	SBV	16.05 ± 0.21	1.31%	23.97 ± 0.26	1.09%
Panel 1	PPRV	16.74 ± 0.24	1.43%	24.58 ± 0.30	1.22%
Panel 1	SPPV-GTPV	17.19 ± 0.23	1.34%	24.96 ± 0.28	1.12%
Panel 2	BDV	16.41 ± 0.13	0.79%	24.35 ± 0.16	0.66%
Panel 2	CAEV	16.58 ± 0.12	0.72%	24.52 ± 0.15	0.61%
Panel 2	Bru	16.26 ± 0.11	0.68%	24.19 ± 0.14	0.58%
Panel 2	*T. gondii*	15.94 ± 0.10	0.63%	23.85 ± 0.13	0.55%
Panel 2	*C. abortus*	16.85 ± 0.12	0.71%	24.69 ± 0.15	0.61%

**Table 8:** Clinical Sample Detection Summary (Panel 1 + Panel 2).

Target	Total Number of Testing Samples	Screening Results of the Developed Multiplex Real-Time PCR Panels	Confirmation Results of WOAH Singleplex PCR/Real Time PCR	Consistency
BTV	620	4 (+)	4 (+)	100%
FMDV	620	13 (+)	13 (+)	100%
SBV	620	0	0	100%
PPRV	620	30 (+)	28 (+)	93.33%
SPPV-GTPV	620	0	0	100%
BDV	620	0	0	100%
CAEV	620	16 (+)	16 (+)	100%
Bru	620	69 (+)	69 (+)	100%
*T. gondii*	620	25 (+)	23 (+)	92.0%
*C. abortus*	620	43 (+)	43 (+)	100%
Total	620	200 (+), 420 (−)	196 (+), 424 (−)	
PPA	98% (196/200)	NPA	99% (420/424)	

**Table 9:** Comparison of multiplex PCR assays for detection of ovine respiratory and reproductive pathogens (2022–2026).

Study (Year)	Pathogens Detected (*n*)	Method	LOD	Sample Matrix	Validation Samples	Detection Time	Key Advantages
Present study (2026)	BTV, FMDV, SBV, PPRV, SPPV/GTPV, BDV, Brucella, *T. gondii*, *C. abortus*, CAEV (10)	2 × 5-plex real-time PCR (freeze-dried)	LOD95:15.8–236 copies/μL (dPCR verified)	Blood, tissue, vaginal swabs, semen, fetal meconium	620 clinical + 111 spiked (731 total)	~2 h	Highest throughput for ovine respiratory + reproductive; freeze-dried stability; absolute quantification by dPCR; multiple matrix validation
Dogan et al. (2025)	Akabane, PPRV, BTV, BVDV, BDV, BoHV, SBV, *C. burnetii*, *L. monocytogenes*, *C. abortus*, *T. gondii*, *N. caninum* (12)	One-run 12-plex qPCR panel	LOD95: 0.45–4.64 log_10_ copies/reaction	Fetal samples (aborted materials)	188 samples (156 ovine + 17 caprine + 15 bovine)	~2 h	Comprehensive coverage of abortive agents; quantitative results; good sensitivity range; overlaps with 6/10 study pathogens
Ke et al. (2024)	*M. ovis*, Pathogenic Leptospira, *C. burnetii*, BTV, PPRV, Brucella, exJSRV, *C. abortus*, ORFV, FMDV, CPV (11)	11-plex real-time PCR (freeze-dried)	40–723 copies/mL (dPCR quantification)	Blood, swabs, pus samples	140 clinical samples	~2 h	Freeze-dried format; room temperature storage; broad pathogen coverage; overlaps with 5/10 study pathogens
Gouvias et al. (2024)	*C. burnetii*, *Chlamydophila* spp., *T. gondii*, *Salmonella* spp., *Brucella* spp., *A. phagocytophilum*, *C. fetus*, *N. caninum*, *L. monocytogenes*, *Leptospira* spp. (10)	10-plex HRM-qPCR		Vaginal swabs	264 samples	~3 h	HRM-based identification; covers major abortive agents; near-perfect agreement with triplex qPCR; overlaps with 2/10 study pathogens
Gao & Fu (2025)	*T. gondii*, *N. caninum*, *E. stiedai*, *G. lamblia*, *T. evansi* (5)	5-plex TaqMan qPCR	1.1 copies/reaction (*T. gondii*)	Various sample types	210 samples	~2 h	Focus on protozoan parasites; TaqMan probe design; high sensitivity; limited pathogen range
Modise et al. (2023)	*Brucella* spp., *C. burnetii*, *Leptospira* spp., *L. monocytogenes* (4)	4-plex qPCR-HRM		DNA samples	216 validation samples	~3 h	HRM discrimination; focused on zoonotic agents; moderate throughput; overlaps with 1/10 study pathogens
Nan et al. (2023)	LSDV, GTPV, SPPV (3)	3-plex real-time PCR	5.41, 27.70, 17.28 copies/μL	Blood, serum, saliva, skin tissue	226 clinical samples	~1.5 h	Capripoxvirus-specific; simple differentiation; high reproducibility (CV < 2.5%); overlaps with 1/10 study pathogens
Das et al. (2022)	CaPV, PaPV, FMDV (3)	3-plex/2-plex real-time PCR	2 CaPV, 7 PaPV, 15 FMDV copies/assay	Clinical samples	103 clinical samples	~1.5–2 h	Differential detection; includes internal control; rule-out approach; overlaps with 2/10 study pathogens

Abbreviations: BTV, Bluetongue virus; FMDV, Foot-and-mouth disease virus; SBV, Schmallenberg virus; PPRV, Peste des petits ruminants virus; SPPV/GTPV, Sheeppox virus/Goatpox virus; BDV, Border disease virus; CAEV, Caprine arthritis-encephalitis virus; LOD, Limit of detection; dPCR, Digital PCR; HRM, High-resolution melting; LSDV, Lumpy skin disease virus; CaPV, Capripoxvirus; PaPV, Parapoxvirus; CV, Coefficient of variation.

## Data Availability

The original contributions presented in this study are included in the article. Further inquiries can be directed to the corresponding author(s).

## References

[1] Quadros D., Milsom A. (2025). Breeds, 124 breeding practices, and health management of sheep flocks in Arkansas, USA. J. Anim. Sci..

[2] Gondard M., Postic L., Garin E., Turpaud M., Vorimore F., Ngwa-Mbot D., Tran M.L., Hoffmann B., Warembourg C., Savini G. (2024). Exceptional Bluetongue virus (BTV) and Epizootic hemorrhagic disease virus (EHDV) circulation in France in 2023. Virus Res..

[3] Fosgate G.T., Oerlemans D.R., Seoke L., Opperman P.A., Lazarus D.D., Gobiye M., Heath L. (2026). Post-vaccination serological status predicts protection against heterologous SAT1 foot-and-mouth disease virus challenge in goats. Prev. Vet. Med..

[4] Udahemuka J.C., Aboge G., Obiero G., Ingabire A., Beeton N., Uwibambe E., Lebea P. (2022). Investigation of foot and mouth disease virus and other animal pathogens in cattle, buffaloes and goats at the interface with Akagera National Park 2017–2020. BMC Vet. Res..

[5] Islam A., Abu Sayeed M., Islam M., Rahman M.K., Islam K.S., Meem H.M., Abedin J., Hossen A., Ahad A., Islam S. (2026). Seroepidemiological Investigation and Risk Factors of Schmallenberg Virus Infection in Sheep and Goats in Bangladesh. Transbound. Emerg. Dis..

[6] Wernike K., Fischer L., Twietmeyer S., Beer M. (2024). Extensive Schmallenberg virus circulation in Germany, 2023. Vet. Res..

[7] Nogales-Altozano P., Martínez-Rodrigo A., Rojas J.M., Sevilla N. (2026). Peste des petits ruminants virus (PPRV) modulates caprine dendritic cell function and induces immunosuppression through IL-10 upregulation. Virulence.

[8] Chahota R., Gupta T., Sharma M., Verma S., Dhar P., Bhardwaj M., Patial V. (2026). Genomic plasticity and phylogeny of sheeppox and goatpox viruses reveal progressive host and terrestrial adaptation. Gene.

[9] Santhamani R., Yogisharadhya R., Venkatesan G., Shivachandra S.B., Pandey A.B., Ramakrishnan M.A. (2014). Molecular characterization of Indian sheeppox and goatpox viruses based on and genes. Virus Genes.

[10] Parrish K., Spiers Z.B., Hazelton M.S., Walker K.H., Duggan E., Graham W., Finlaison D.S., Kirkland P.D. (2026). Large-scale reproductive loss in sheep due to Border disease virus infection, New South Wales, Australia. Aust. Vet. J..

[11] Alshekh K.A., Shahlol A.M., Ben Mostafa K.K., Othman A.A., Othman A.A., Hiblu M.A., Abouzeed Y.M., Daw M.A., Ahmed M.O. (2024). Seroprevalence of brucellosis in sheep and goats from Al Jufrah district in Libya. Pan Afr. Med. J..

[12] Brunetti R., Ottaiano M., Fordellone M., Chiodini P., Signoriello S., Gargano F., De Massis F., Baldi L., De Carlo E. (2023). Risk Factors for the Spread of Brucellosis in Sheep and Goats in the Campania Region in the Years 2015–2020. Microorganisms.

[13] Issad N.A., Cherif A.M., Mebkhout F., Alilleche K., Dadda A., Abdelouahed K., Khelef D. (2026). First report of molecular and histopathological detection of in aborted fetal goat myocardium in Algeria with associated risk factors. Parasitol. Int..

[14] Boukhalfa N., Douifi M. (2025). Prevalence and risk factors of infection in small ruminants from North-Central Algeria. Comp. Immunol. Microbiol. Infect. Dis..

[15] Borel N., Longbottom D., Greub G., Albini S., Vanrompay D., Laroucau K. (2025). Zoonotic infections due to avian *Chlamydia abortus*: What are we missing?. Lancet Microbe.

[16] Bárdos K., Máté M., Veres K., Lang Z.L., Bertoni G., Abril C.E., Stuen S., Petkevicius S., Mickiewicz M., Czopowicz M. (2025). Bayesian Estimation of the True Prevalence of Caprine Arthritis Encephalitis in Hungarian Goat Herds. Viruses.

[17] Yang J., Li D.D., Wang J., Zhang R., Li J.M. (2022). Design, optimization, and application of multiplex rRT-PCR in the detection of respiratory viruses. Crit. Rev. Clin. Lab. Sci..

[18] Hawkins S.F.C., Guest P.C. (2017). Multiplex Analyses Using Real-Time Quantitative PCR. Multiplex Biomarker Techniques: Methods in Molecular Biology.

[19] Li W.X., Li J.W., Li R., Chen G.J., Shum P.P., Zou Y., Chen R.T., Dong Y.K., Xu X.C. (2026). Breaking the Bottleneck in Limit of Detection of Surface Refractive Index Sensing by Harnessing Meta-Waveguide Microring Resonators. Adv. Sci..

[20] Xin J.G., Ji X.C., Song Z.G., Zuo W.D., Yin S.L., Peng Y., Ren M., Ai J., Han D.G. (2026). Bluetongue in China: Current Status of Viruses, Vectors, Detection Methods, and Vaccines. Transbound. Emerg. Dis..

[21] De Regge N., Deblauwe I., Deken R., Vantieghem P., Madder M., Geysen D., Smeets F., Losson B., van den Berg T., Cay A.B. (2012). Detection of Schmallenberg virus in different Culicoides spp. by real-time RT-PCR. Transbound. Emerg. Dis..

[22] Hole K., Nfon C. (2019). Foot-and-mouth disease virus detection on a handheld real-time polymerase chain reaction platform. Transbound. Emerg. Dis..

[23] Mahapatra M., Howson E., Fowler V., Batten C., Flannery J., Selvaraj M., Parida S. (2019). Rapid Detection of Peste des Petits Ruminants Virus (PPRV) Nucleic Acid Using a Novel Low-Cost Reverse Transcription Loop-Mediated Isothermal Amplification (RT-LAMP) Assay for Future Use in Nascent PPR Eradication Programme. Viruses.

[24] Balinsky C.A., Delhon G., Smoliga G., Prarat M., French R.A., Geary S.J., Rock D.L., Rodriguez L.L. (2008). Rapid preclinical detection of Sheeppox virus by a real-time fluorescent PCR assay. J. Clin. Microbiol..

[25] Legesse A., Mekuriaw A., Gelaye E., Abayneh T., Getachew B., Weldemedhin W., Tesgera T., Deresse G., Birhanu K. (2023). Comparative evaluation of RBPT, I-ELISA, and CFT for the diagnosis of brucellosis and PCR detection of Brucella species from Ethiopian sheep, goats, and cattle sera. BMC Microbiol..

[26] Sah R.P., Dey A.R., Rahman A.K.M.A., Alam M.Z., Talukder M.H. (2019). Molecular detection of from aborted fetuses of sheep, goats and cattle in Bangladesh. Vet. Parasitol. Reg. Stud. Rep..

[27] Rosamilia A., Grattarola C., Caruso C., Peletto S., Gobbi E., Tarello V., Caroggio P., Dondo A., Masoero L., Acutis P.L. (2014). Detection of border disease virus (BDV) genotype 3 in Italian goat herds. Vet. J..

[28] Cruz J.C.M., Gouveia A.M.G., Souza K.C., Braz G.F., Teixeira B.M., Heinemann M.B., Leite R.C., Reis J.K.P., Pinheiro R.R., Andrioli A. (2009). Caprine arthritis-encephalitis virus (CAEV) detection in semen of endangered goat breeds by nested polymerase chain reaction. Small Rumin. Res..

[29] Spencer S., Thompson M.G., Flannery B., Fry A. (2019). Comparison of Respiratory Specimen Collection Methods for Detection of Influenza Virus Infection by Reverse Transcription-PCR: A Literature Review. J. Clin. Microbiol..

